# Interaction of Surface Energy Components between Solid and Liquid on Wettability, and Its Application to Textile Anti-Wetting Finish

**DOI:** 10.3390/polym11030498

**Published:** 2019-03-14

**Authors:** Kwanwoo Song, Jinwook Lee, Seong-O Choi, Jooyoun Kim

**Affiliations:** 1 Department of Textiles, Merchandising and Fashion Design, Seoul National University, Seoul 08826, Korea; skwsong1@snu.ac.kr (K.S.); shop0319@snu.ac.kr (J.L.); 2 Department of Chemical Education, Seoul National University, Seoul 08826, Korea; 3 Department of Anatomy and Physiology, Kansas State University, Manhattan, KS 66506, USA; sochoi@ksu.edu; 4 Nanotechnology Innovation Center of Kansas State, Kansas State University, Manhattan, KS 66506, USA; 5 Research Institute of Human Ecology, Seoul National University, Seoul 08826, Korea

**Keywords:** surface energy, dispersive, polar, anti-wetting, roughness, nonwoven, etching, contact angle, thermodynamics

## Abstract

With various options of anti-wetting finish methods, this study intends to provide basic information that can be applied in selecting a relevant anti-wetting chemical to grant protection from spreading of liquids with different surface energy profiles. With such an aim, the anti-wetting effectiveness of fluorinated coating and silane coating was investigated for liquids having different surface energy components, water (WA), methylene iodide (MI) and formamide (FA). The wetting thermodynamics was experimentally investigated by analyzing dispersive and polar component surface energies of solids and liquids. The role of surface roughness in wettability was examined for fibrous nonwoven substrates that have varied surface roughness. The presence of roughness enhanced the anti-wetting performance of the anti-wetting treated surfaces. While the effectiveness of different anti-wetting treatments was varied depending on the liquid polarities, the distinction of different treatments was less apparent for the roughened fibrous surfaces than the film surfaces. This study provides experimental validation of wetting thermodynamics and the practical interpretation of anti-wetting finishing.

## 1. Introduction

Surface wettability plays an important role in many functional applications [1] such as superhydrophobic self-cleaning materials [2], anti-fouling surfaces [3,4], liquid separation [2,5,6,7], corrosion protection [8], and fluid control [9,10,11]. With various industrial applicability, strategies for modifying surface wettability have been immensely studied [9,10,12,13,14,15,16,17,18]. One of the common design strategies for making an anti-wetting surface is to lower the surface energy and to implement the surface roughness [9,13,19,20,21,22,23,24]. While the material’s intrinsic wettability depends primarily on the surface energy, the role of surface roughness becomes critical when fabricating extremely anti-wetting surfaces [9,22,25]. Recent findings have shown that forming surface roughness in re-entrant structures can produce an inherently unwetting surface [25,26,27,28], regardless of the surface energy. Surface wettability is generally represented by the measure of contact angle (CA) and shedding angle (ShA). Commonly accepted criteria for superhydrophobic surface are CA > 150° and ShA < 10° [19,20].

Traditionally Young’s equation [29] explains the solid–liquid interactions in wetting using the overall surface energies of solids and liquids, assuming a smooth and flat solid surface. The interfacial energy of solids and liquids depends on the dispersive and polar components of the surface energy of individual solid and liquid. From this relationship, the wettability is generally reduced when the overall surface energy of the solid surface is lowered. Early studies on anti-wetting surface modification have been focused on novel anti-wetting chemicals with a low surface energy or durable treatment methods [5,30,31,32]. Fluorinated compounds have been extensively applied in anti-wetting finish methods due to their very low surface energy [19,20,31,33,34]. Recently the environmental concern has been raised on the use of perfluorinated compounds having chain lengths of eight carbons (C8) or longer [35], and the alternatives to C8 perfluorinated chemicals, including silane with long carbon chains, are being explored [36,37,38]. 

The overall surface energy is the sum of the polar and dispersive components of surface energy [39,40]. Different anti-wetting agents with similar overall surface energy; however, could have distinct surface energy components, which would affect their interfacial interaction with liquids and thereby would show different wettability. Due to an increasing interest in anti-wetting materials, extensive studies are available for anti-wetting fabrication, most of which are focused on surface designs to maximize repellency or anti-wetting [5,6,26,27,31,41,42]. However, the interplay between the anti-wetting surface and liquids has rarely been discussed although the wetting thermodynamics would be different depending on the surface energy profiles of the anti-wetting agents and the wetting liquid. 

While the reduction of surface energy is the primary means of anti-wetting fabrication, it is often not sufficient to attain extreme repellency only by modifying the surface energy of solid surfaces. Thus, the implementation of surface roughness becomes significant in achieving the high level of anti-wetting property [43,44]. When an anti-wetting coating is applied to fabrics, the surface usually displays a higher level of anti-wetting than flat film surfaces, due to the presence of roughness formed by the microscale fibers and yarns [19,20,32,33,45]. With the additional roughness formed by the particle deposition [38,45] or the surface etching [36,46,47], the fabric surface can further enhance the anti-wetting property [9,22,25,26]. 

This study aims at providing the experimental investigation on the solid–liquid interactions, using the concept of surface energy components and the related thermodynamics. To investigate interface interactions, polymeric surfaces were treated with two different anti-wetting chemicals, fluorinated and silane compounds, and the wettability was measured with three different liquids. Anti-wetting coating was applied either by plasma enhanced chemical vapor deposition (PECVD) using C_4_F_8_ gas, or by physical vapor deposition using dodecyltrimethoxy silane (DTMS). The tested liquids were water (WA), formamide (FA), and methylene iodide (MI), which have different surface energy components. To examine the interplay between the surface energy components, dispersive and polar components of the tested surfaces and liquids were analyzed. The spreading parameter of the liquid on the solid surface was calculated using two components of surface energy [39]. 

The study also intends to examine the interplay of roughness and surface energy on the wetting of liquids on fibrous surfaces. Surfaces with different roughness, including flat film, spunlace (SL) nonwoven, spunbond (SB) nonwoven, and SB with alkali-etching, were examined for anti-wetting properties. The surface profiles were observed by scanning electron microscopy (SEM) and atomic force microscopy (AFM). 

Anti-wetting fabrics can be applied to a protective garment that needs protection against exposure to hazardous liquids. To achieve the anti-wetting performance to a particular liquid, understanding the interfacial interaction between the solid and liquid is significant. The emphasis of this study lies in the experimental validation of wetting thermodynamics, and its extension to the practical application of textile finish. The result of this study would be useful in choosing a proper anti-wetting finishing agent for the protection against a particular liquid with known surface energy components or polarity.

## 2. Materials and Methods 

### 2.1. Materials

Sodium hydroxide (NaOH) was purchased from Junsei Chemical Co. (Tokyo, Japan). Octafluorocyclobutane (C_4_F_8_) gas was obtained from Union Gas (Yongin, South Korea). Chemicals, including dodecyltrimethoxysilane (DTMS), methylene iodide (MI), and formamide (FA), were purchased from Sigma Aldrich (St. Louis, MO, USA). Three different polymeric substrates, cellulose (Cel), polypropylene (PP), and polyethylene terephthalate (PET), were used for surface wetting analysis. Those polymers have different surface energy profiles and are commonly used as nonwoven materials for various applications. Cellulose and PET films were purchased from Goodfellow (Huntingdon, UK). PP films were prepared using PP resin obtained from SK Global Chemical Co., Ltd. (Seoul, South Korea). Cellulose and PET nonwovens were obtained from Korea Institute of Industrial Technology (KITECH, Cheonan, South Korea). The specifications of films and nonwovens used in this study are shown in Table 1.

### 2.2. Anti-Wetting Coating

For hydrophobic coating, two different methods were used; plasma enhanced chemical vapor deposition (PECVD) and physical vapor deposition (PVD). For PECVD, a gas flow of C_4_F_8_ was provided with 100 sccm for 25 min at 160 W in Covance plasma system (Femto Science, Hwaseong, South Korea) for the polymerization of a fluorinated compound on the substrate. For the PVD method, 10 mL of dodecyltrimethoxysilane (DTMS) was vaporized in a vacuum oven (oven inner volume ~0.13 m^3^, LVO-2051P, Daehan Labtech, Namyangju, South Korea) at 120 °C for 3 h to coat the substrate. 

### 2.3. Alkali Etching

Nanoscale roughness was created on PET surfaces utilizing the alkali hydrolysis reaction of polyester [48]. For this treatment, a 30% (*w*/*w*) NaOH solution was prepared with distilled water, and a 10 cm × 10 cm PET nonwoven specimen was immersed in 50 mL solution at 80 °C for 60 min. The alkali treated specimens were rinsed with distilled water till pH reached 7, and dried at 80 °C for 2 min. The weight loss after alkali hydrolysis was measured to be ~50%. Alkali-etched PET spunbond nonwoven samples were coated with DTMS or C_4_F_8_ as previously described for hydrophobic modification. The specimens used in this study are described in Table 2.

### 2.4. Wettability

For wetting analysis, static contact angle (CA) and shedding angle (ShA) of liquids on surfaces were measured using a contact angle analyzer (SmartDrop Lab, Femtobiomed Inc., Seongnam, South Korea). Contact angles of distilled water (WA), methylene iodide (MI), and formamide (FA) liquids were measured to examine the wettability of surfaces by liquids with different surface energy components. The surface energy components of different liquids used in this study are listed in Table 3. The ratio of polarity to the overall surface energy of solid (P_S_) and liquid (P_L_) was calculated (P_L_ in Table 3, P_S_ in Table 4).

For CA measurement, 2.5 ± 0.5 µL of liquid drops was placed on a surface, and CA was measured within 1 s after the droplet settlement. At least five measurements were obtained from different spots of the surfaces, and the mean value was used for analysis. For ShA measurement, 12.5 µL of liquid was dropped vertically from 1 cm above the sample that was laid on a tilted stage. The lowest angle at which the drop starts to roll more than 2 cm on the surface was recorded as ShA, and the mean value of five measurements was used for analysis. 

### 2.5. Surface Energy of Solid Surface

Surface energy with its dispersive and polar components for the solid surfaces was estimated using the Owens–Wendt model [40]. With the known surface energy components of liquids and by measuring the contact angle on a flat surface, the surface energy components of solid substrates can be calculated, as shown in Equations (1)–(4) [40]. For the calculation, contact angles of WA and MI were measured on flat film surfaces. The surface energy of the nonwoven surface was regarded as the same as that of the respective film.

(1) $$\gamma_{SL}=\gamma_{S}+\gamma_{L}-2\sqrt{\gamma_{S}^{d}\cdot \gamma_{L}^{d}}-2\sqrt{\gamma_{S}^{p}\cdot \gamma_{L}^{p}}$$

(2) $$\gamma_{S}=\gamma_{SL}+\gamma_{L}\;\cos\theta$$

(3) $$\gamma_{L}\left(1+cos\theta \right)=2\sqrt{\gamma_{S}^{d}\cdot \gamma_{L}^{d}}-2\sqrt{\gamma_{S}^{p}\cdot \gamma_{L}^{p}}$$

(4) $$\gamma_{S}=\gamma_{S}^{d}+\gamma_{S}^{p}$$

- θ: contact angle of liquid on solid surface
- $\gamma_{SL}$: interfacial energy between solid and liquid
- $\gamma_{S}$: surface energy of solid
- $\gamma_{S}^{d}$: dispersive component surface energy of solid
- $\gamma_{S}^{p}$: polar component surface energy of solid
- $\gamma_{L}$: surface energy of liquid
- $\gamma_{L}^{d}$: dispersive component surface energy of liquid
- $\gamma_{L}^{p}$: polar component surface energy of liquid

### 2.6. Surface Chemistry and Morphology

The surface chemistry of substrates in terms of atomic concentration (%) was analyzed using X-ray photoelectron spectroscopy (XPS, Axis Supra^TM^, Kratos Analytical, Manchester, UK), to verify the presence of coating compounds on the surfaces. The surface morphology of the samples was observed using a field-emission scanning electron microscope (FE-SEM, Supra 55VP, Carl Zeiss, Jena, Germany), with prior Pt coating (~10 nm) at 30 mA for 200 s using a sputter coater (EM ACE200, Leica, Wetzlar, Germany). For observation of surface roughness, an atomic force microscope (AFM, NX-10, Park Systems, Suwon, South Korea) was used with non-contact mode, and the root mean square of height (RMS) was measured as representative roughness information. 

## 3. Results and Discussion

### 3.1. Wettability of Flat Surfaces

#### 3.1.1. Surface Energy Modification with Hydrophobic Treatment

**Figure 1:**
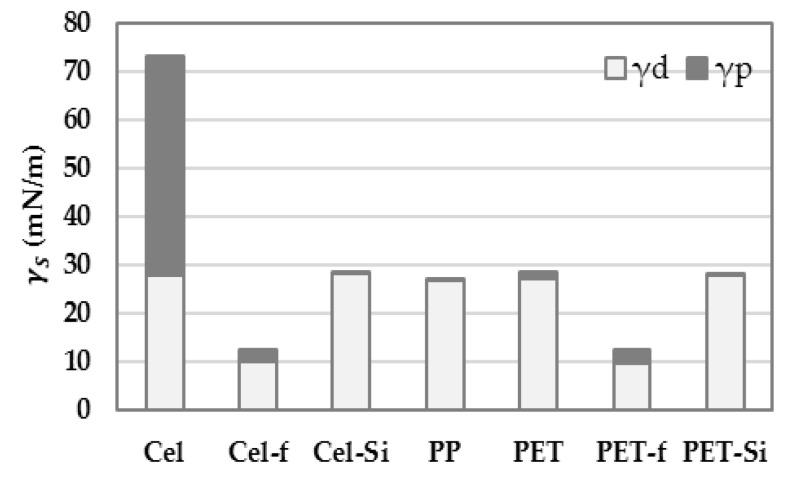
Surface energy components of solid surfaces.

Polymeric substrates of cellulose (Cel) and polyethylene terephthalate (PET) were treated with silane (DTMS) and fluorinated (C_4_F_8_) compounds for anti-wetting surface modification. Polypropylene (PP) consisting completely of hydrocarbons was compared with those surfaces. For the substrates, surface energy and its components were calculated by measuring the CA of water (WA) and methylene iodide (MI). The P_S_ (polar ratio) of solid was calculated by the ratio of the polar component to the overall surface energy. The CA measurements and the surface energy estimates are shown in Table 4 and Figure 1.

For both Cel and PET substrates, surface coating with DTMS and C_4_F_8_ reduced the overall surface energy, down to about 28 mN/m and 12 mN/m, respectively. With the silane coating, the dispersive component was significantly reduced for both Cel and PET and the Ps of surface energy reached 0.006–0.015. On the contrary, the fluorinated coating produced higher Ps (0.18–0.22) compared to the silane coating, while the overall surface energy of fluorinated surface (12 mN/m) was considerably lower than that of DTMS coated surface (28 mN/m). The surface energy of PP, which consists of hydrocarbons, was calculated to be about 27 mN/m, most of which is contributed by the dispersive component (>99%). The contribution of each polar and dispersive component to the overall surface energy is presented in Figure 1.

The change of surface chemistry after the coating process was examined by XPS (Table 5). It was observed that the atomic concentration (%) of O and Si in the DTMS-treated surfaces was significantly larger than other surfaces. The deposition of DTMS was more apparent for the Cel samples than for the PET samples, probably due to the higher affinity of silane compounds to –OH groups of Cel substrate. The C_4_F_8_-treated surfaces showed a significant amount of F. The result confirms that the decrease of surface energy is attributed to the presence of coated chemicals.

#### 3.1.2. Solid–Liquid Interaction on Wettability

Wettability is the result of liquid–solid interactions, and cosθ can be expressed as the relationship between surface energies of a liquid and solid (Equations (5) and (6), where θ is the contact angle and Φ is the interfacial function in the range of 0 to 1 [50].

(5) $$\gamma_{SL}=\gamma_{S}+\gamma_{L}-2\Phi \sqrt{\gamma_{S\cdot }\gamma_{L}}$$

(6) $$\cos\theta =2\Phi \sqrt{\frac{\gamma_{S}}{\gamma_{L}}}-1$$

**Figure 2:**
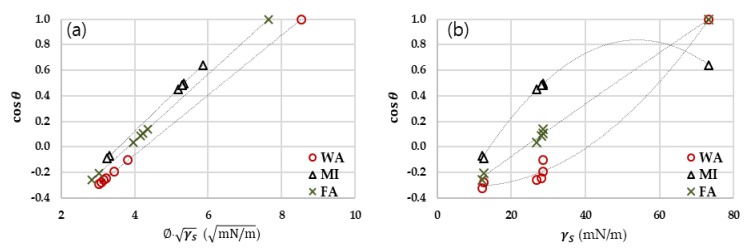
Wettability with different surface energy surfaces. (**a**) cosθ with different Φ$\sqrt{\gamma_{S}}$; (**b**) cosθ with different $\gamma_{S}$.

In Equation (6), cosθ would be proportional to Φ $\sqrt{\gamma_{S}}$, as shown in Figure 2a. From Figure 2b, while cosθ increased with an increase in the overall surface energy of solid, the relationship between cosθ and $\gamma_{S}$ does not show a clear tendency. The result confirms the fact that the wetting cannot be accurately predicted by a single factor of solid surface energy. As the wetting is the result of the solid–liquid interactions, surface energies of both liquids and solids need to be counted in predicting the wettability. From Equation (6), the interfacial function Φ is proportional to cosθ, and the complete wetting state (cosθ of 1) corresponds to the maximum Φ. However, cosθ is not perfectly correlated with Φ alone. Instead, the solid–liquid interplay in terms of their surface energy components needs to be considered when predicting the surface wetting. 

#### 3.1.3. Interplay of Surface Energy Components on Wetting 

**Figure 3:**
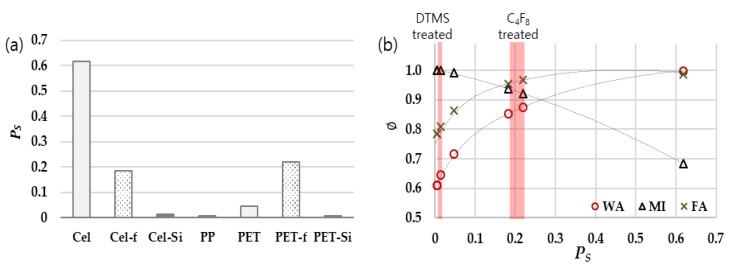
Surface polarity influencing wetting. (**a**) Percentage of the polar contribution to the overall surface energy of solids; (**b**) Interfacial function with % polarity.

As the wetting thermodynamics is influenced by the dispersive and polar components of surface energy, different liquids with the same overall surface energy can exhibit different wettability on a solid surface if the liquids have different surface energy components. The surface energy components of a phase can be reflected in the polar ratio, and the Ps for different substrates is shown in Figure 3a. For PP and the silane-treated surfaces, the polar component contribution to the overall surface energy was close to zero due to the dominant arrangement of nonpolar hydrocarbons on the surfaces. While the fluorinated surfaces had the lowest overall surface energy, its Ps was higher than that of PP and the silane-treated surfaces, probably due to the polarity of fluorine.

To examine the interplay between surface energy components of solids and liquids on wetting, Equations (1) and (5) were combined to produce Equations (7) and (8). By differentiating Equation (8), Equation (9) is derived.

(7) $$\sqrt{\gamma_{S}^{d}\cdot \gamma_{L}^{d}}+\sqrt{\gamma_{S}^{p}\cdot \gamma_{L}^{p}}=\Phi \sqrt{\gamma_{S\cdot }\gamma_{L}}$$

(8) $$\Phi =\sqrt{\frac{\gamma_{S}^{d}\cdot \gamma_{L}^{d}}{\gamma_{S\cdot }\gamma_{L}}}+\sqrt{\frac{\gamma_{S}^{p}\cdot \gamma_{L}^{p}}{\gamma_{S\cdot }\gamma_{L}}}=\sqrt{\left(1-P_{S}\right)\left(1-P_{L}\right)}+\sqrt{P_{S}P_{L}}$$

(9) $$\frac{d}{{\mathrm{dP}}_{S}}=-\frac{1}{2}\sqrt{\frac{\left(1-P_{L}\right)}{\left(1-P_{S}\right)}}+\frac{1}{2}\sqrt{\frac{P_{L}}{P_{S}}}$$

(10) $$\cos\theta =2\left[\sqrt{\left(1-P_{S}\right)\left(1-P_{L}\right)}+\sqrt{P_{S}P_{L}}\right]\;\left[\sqrt{\frac{\gamma_{S}}{\gamma_{L}}}-1\right]$$

when $\frac{d\Phi }{{\mathrm{dP}}_{S}}$ is zero (when P_S_ = P_L_), Φ has the maximum value of 1. P_L_ values for liquids are: 0.7 for water; 0 for MI; and 0.46 for FA. Using those P_L_ values, the interfacial function, Φ can be calculated from Equation (8), resulting in: WA, Φ = $\sqrt{0.3\left(1-P_{S}\right)}+\sqrt{0.7P_{S}}$; MI, $\Phi =\sqrt{\left(1-P_{S}\right)}$; FA, $\Phi =\sqrt{0.54\left(1-P_{S}\right)}+\sqrt{0.46P_{S}}$. The maximum Φ of 1 would appear when P_S_ values are 0.7, 0, and 0.46 for WA, MI, and FA, respectively. In Figure 3b, Ps of Cel_-Si_, PET_-Si_, Cel_-f_, and PET_-f_ were 1.47, 0.61, 18.4, and 21.9, respectively. When Φ was plotted as a function of P_S_ of various solids, the maximum values of Φ appear approximately at the Ps values of 0.7 for WA, 0.46 for FA, and 0 for MI.

It is noteworthy that the interfacial function Φ evolves differently depending on the polarity of liquids (P_L_). For MI, which is a nonpolar liquid, Φ increases as P_S_ decreases. For polar liquids, such as WA and FA, Φ shows the maximum value at certain polarity of solids; maximum Φ appears at Ps of 0.7 for WA and Ps of 0.46 for FA. Based on the result, it is inferred that the reduction of polarity of solid is effective for anti-wetting of a polar liquid, while the reduction of dispersive surface energy is effective for anti-wetting of nonpolar liquid. Thus, the effect of anti-wetting for a specific repellent coating would be varied for different liquids, and the wettability or anti-wettability against a certain liquid can be estimated using the surface polarity, Ps. 

**Figure 4:**
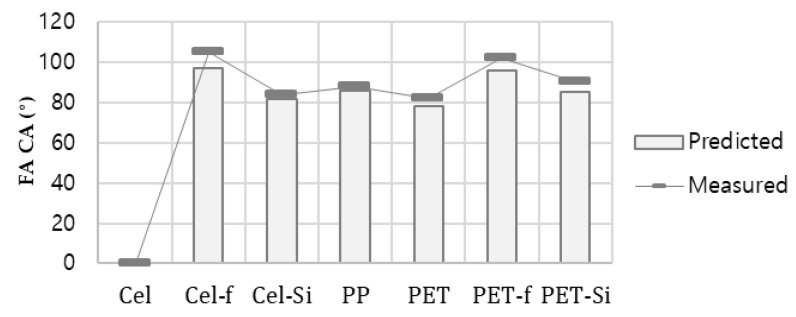
Contact angle of formamide (FA), actual measurement and estimation from Equation (10).

Combining Equations (6) and (8), Equation (10) is derived, which can be used in estimating CA of liquids on different surfaces. In Figure 4, the predicted (from Equation (10)) and measured CAs for FA on surfaces with various polar ratio are presented. While the discrepancy exists between the measured and predicted values (<6°), the wettability could be predicted to some extent. 

Understanding that the wetting is determined by the interplay between surface energy components of solids and liquids, the thermodynamics for spreading was examined. Spreading occurs when a liquid wets a solid surface in the air. The spreading parameter, S, is defined by the work of adhesion (solid–liquid interaction) subtracted by the work of cohesion (liquid–liquid interaction), and S can be expressed as Equation (11) [39]. Combining Equations (1) and (11), Equation (12) is derived, where the spreading parameter, S, is expressed by the two surface energy components of a solid and liquid. If S ≥ 0, the liquid completely wets the surface in order to lower the surface energy; a lower S represents a lower wettability (or lower spreading) as a result of the lower interaction between the solid and liquid.

(11) $$S=\gamma_{S}-(\gamma_{SL}+\gamma_{L})$$

(12) $$S=2(\sqrt{\gamma_{S}^{d}\cdot \gamma_{L}^{d}}+\sqrt{\gamma_{S}^{p}\cdot \gamma_{L}^{p}}-\gamma_{L})$$

where S is the spreading parameter.

**Figure 5:**
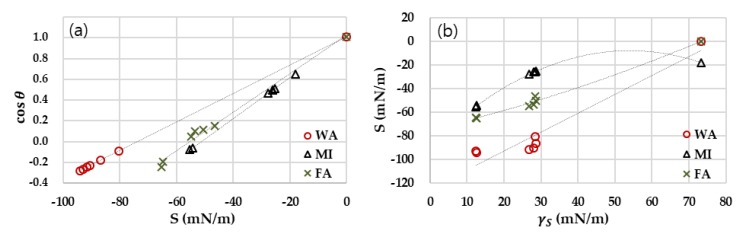
Spreading parameter for surface energy and wettability. (**a**) Plots of cosθ with spreading parameter; (**b**) Spreading parameter with different surface energy of the solid.

The experimental investigation of the spreading parameter is presented in Figure 5. The relationship between S and cosθ is linearly correlated (Figure 5a), and the complete wetting (cosθ = 1) occurs at S = 0. The slopes of the plots decrease when the polarity of liquid increases; WA showed the lowest slope and MI showed the highest slope among the tested liquids. From Figure 5b, S increases when $\gamma_{S}$ increases, showing the general tendency of favorable wetting of solids with higher surface energy. However, the rate of increase in S was not consistent; for nonpolar liquid, the rate of increase for S declines as $\gamma_{S}$ increases, while, for a polar liquid, the rate of increase rises as $\gamma_{S}$ becomes larger. For FA, of which polarity is about 46%, the rate of increase for S was consistent regardless of $\gamma_{S}$, displaying the linear relationship between the S and $\gamma_{S}$. 

The results of this study can be used as a selection guide of anti-wetting chemicals by the type of liquids that need to be unwet. For example, either silane or fluorinated compound would be sufficient to grant the anti-wetting property against water; however, if the solid needs to be protected from wetting against a nonpolar liquid such as MI, then the fluorinated coating agent that has lower dispersive component compared to silane coating would be preferable for better anti-wetting performance. The results so far showed the experimental validation of wetting thermodynamics, employing different anti-wetting treatments on flat surfaces. In Section 3.2, the role of surface roughness in the anti-wetting property is further examined.

### 3.2. Wetting of Fibrous Surfaces

**Figure 6:**
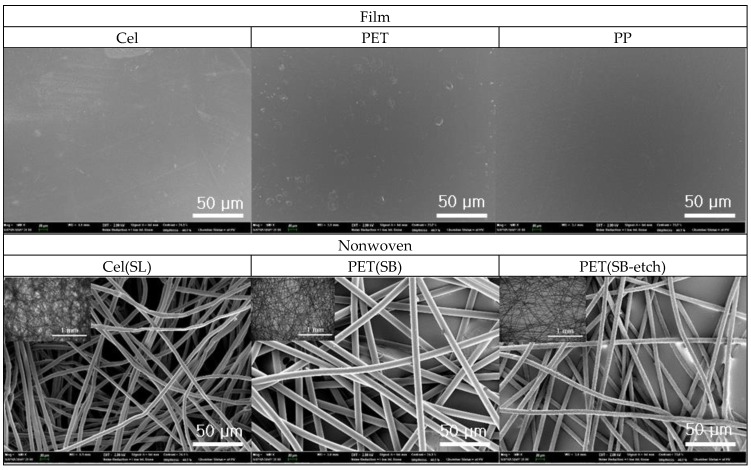

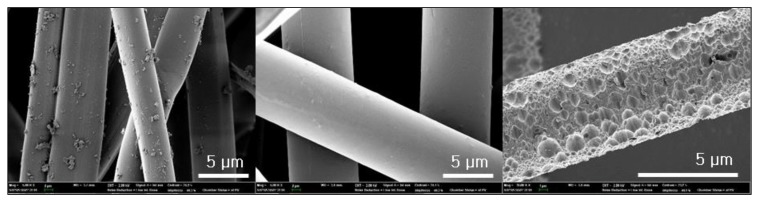
Scanning electron microscopy (SEM) images of surfaces. Inserts are optical microscope images with the scale bar of 1 mm.

The wettability is the result of the interplay between surface energy and surface roughness. Therefore, the effect of surface roughness in addition to solid’s surface energy was further examined using fibrous nonwoven substrates. The nonwoven types used in this study included Cel(SL) and PET(SB), which are commonly used in disposable applications. As the fibers of the nonwoven surface would form limited surface roughness in microscale, the PET(SB) samples underwent alkali hydrolysis to create nanoscale roughness. The morphology of the substrates with different roughness profiles was observed by SEM as shown in Figure 6. The Cel(SL) samples had unknown particles on the surfaces even after the surface was cleaned; the compositions of the particles were found to be C and O, and the particles were thought to come from the nonwoven process aids. Due to the presence of such particles, Cel(SL) had additional roughness. The Cel(SL) had smaller fibers with denser layers than PET(SB), producing relatively smaller-scale roughness; the fiber sizes of Cel(SL) and PET(SB) were rather uniform, which were measured to be ~9.6 ± 1.2 μm and ~17.3 ± 0.5 μm, respectively for Cel(SL) and PET(SB). The roughness between SL and SB surfaces, however, could not be quantified by SEM.

**Figure 7:**
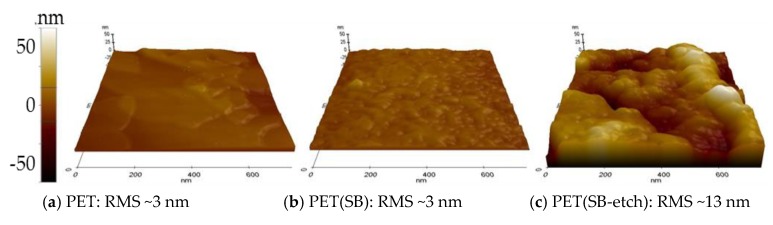
Surface roughness observed by atomic force microscopy (AFM). (**a**) Polyethylene terephthalate (PET) film; (**b**) PET spunbond fabric; (**c**) alkali-etched PET spunbond fabric.

AFM can measure the variation of height profiles, in a value of root mean squared height (RMS). However, due to the limited z-scan range (≤4 μm), the RMS information was available only for comparing the submicron scale roughness. The AFM scanning was carried out across the samples (0.8 μm × 0.8 μm), and the RMS values of PET film, PET SB, and alkali-etched PET SB were compared (Figure 7). The RMS value of PET(SB) was not different from that of PET film as the scanning was conducted on the small area of the topmost fiber surface. The PET(SB-etch), however, showed significantly large RMS (~13 nm), confirming the presence of nanoscale roughness in PET(SB-etch). 

**Figure 8:**
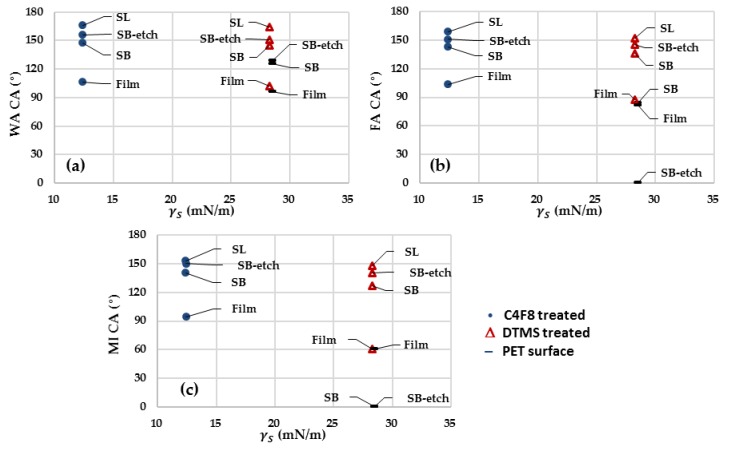
Wettability of substrates with different surface energy and roughness profiles.

The CA and ShA measurements for the varied substrates are shown in Table 6 and Figure 8. For all fibrous surfaces, the presence of roughness considerably increased the CAs, effectively enhancing the anti-wetting property. Particularly, the surfaces with the nanoscale roughness, Cel(SL)_-f_, Cel(SL)_-Si_, PET(SB-etch)_-f_, and PET(SB-etch)_-Si_, displayed the superhydrophobic property with WA CA ≥ 150° and WA ShA ≤ ~10°. The result clearly shows the critical role of nanoscale roughness on the anti-wetting property. 

For further analysis, the Cassie–Baxter state [51] was assumed for the surfaces that displayed the water CA ≥ 147° and ShA ≤ 15°, which were Cel(SL)_-f,_ Cel(SL)_-Si_, PET(SB)_-f_, PET(SB-etch)_-f_, and PET(SB-etch)_-Si_. If it is assumed that the liquid drop contacts only the topmost surface of the roughened substrate, instead of intruding into the protrusions, the Cassie–Baxter model [51] can be simplified as Equation (13) [19]. In the equation, the solid fraction, f, can be roughly estimated from the measured CA’s of fibrous surfaces ($\theta_{\mathrm{CB}}$) and those of flat film surfaces ($\theta_{Y}$). The estimated f, which is the topmost area fraction of the unit area, were 0.045, 0.23, and 0.142 for the Cel SL, PET SB, and etched PET SB surfaces, respectively. The result showed that the cellulose spunlace had the least area wet by the liquid, probably due to the irregular depth of fibers and fine particles on the surface.

(13) $${\cos\theta }_{\mathrm{CB}}=f\cdot \left({\cos\theta }_{Y}+1\right)-1$$

- $\theta_{\mathrm{CB}}$: contact angle in the Cassie–Baxter state
- $\theta_{Y}$: contact angle in Young’s model in Equation (2) (flat surface)
- f: solid fraction in the Cassie–Baxter model, which is the fraction of wetted surface area to the overall projected surface area.

From Table 6, the film surfaces treated with DTMS and C_4_F_8_ showed a similar level of anti-wetting performance for water, while the surface treated with C_4_F_8_ showed the considerably enhanced anti-wetting performance for MI. The discrepancy of anti-wetting performance between the DTMS and C_4_F_8_ treatment became larger as the polarity of liquid decreased (P_L_ is WA > FA > MI). As was discussed earlier, the results demonstrate that the anti-wetting agent needs to be properly selected based on the property of the applied liquid. The overall tendency of the anti-wetting performance of fibrous surfaces was similar to that of film surfaces; however, the fibrous surfaces did not show dramatic performance distinction between the DTMS and C_4_F_8_ treatment for different liquids compared with the films. It can be inferred that the decreased wetted area (with the concept of solid fraction, f) would lower the interaction of surface energy between the solid and liquid, thereby reducing the influence of different anti-wetting agents. 

The findings of this study can be applied to anti-wetting finish methods, where a proper selection of anti-wetting agent needs to be made for the protection from spreading of liquids. While it is important to attain a low level of overall surface energy in reducing the wettability, the component profiles of surface energy also play a critical role in liquid spreading on surfaces. Textile materials with microscale roughness would exhibit enhanced anti-wetting compared to flat surfaces, if the same type of anti-wetting chemicals are applied. To further improve the anti-wetting performance, implementation of submicron scale roughness would be beneficial. For the materials that need adequate protection against the exposure of non-polar liquid (such as MI), an anti-wetting treatment with a low dispersive component may be effective. For the materials that need to be protected from the spreading of a polar liquid, anti-wetting chemicals with a low level of the dispersive component may be effective. 

## 4. Conclusions

With the increased awareness of sustainable finishing, various options of anti-wetting finish methods are being explored. This study intends to provide basic information that can be used in selecting an effective anti-wetting agent for protection against the spreading of liquids with particular surface energy profiles. With such an aim, the anti-wetting effectiveness of fluorinated and silane coating was investigated using the liquids having different surface energy components: water (WA), methylene iodide (MI), and formamide (FA). The wetting thermodynamics was experimentally investigated by analyzing two components of surface energies for solids and liquids, with the theoretical discussion on the interplay of components on wettability. The effectiveness of different anti-wetting treatments was varied for liquids with different polarities.

The role of surface roughness in wettability was examined for fibrous nonwoven substrates that have various levels of roughness. The presence of nanoscale roughness resulted from nanoparticles or alkali etching was beneficial for attaining a high level of anti-wetting performance. The difference of anti-wetting performance between the treatments was less apparent for the roughened surfaces than for the flat film surfaces. The findings of this study can be used in selecting proper anti-wetting chemicals to grant protection from spreading of specific liquids. While it is important to attain a low level of overall surface energy for reducing wettability, the surface energy components also play a critical role in liquid spreading on surfaces. For example, an anti-wetting treatment with a low dispersive component may be desirable for a material that needs protection from the exposure of non-polar liquids, such as MI. This study provides experimental validation of wetting thermodynamics and practical interpretation of anti-wetting finishing.

## Figures and Tables

**Table 1:** Film and nonwoven substrates used.

Substrate	Polymer	Process	Thickness (mm)	Basis Weight (g/m^2^)
Film	Cellulose	Casting	0.3	45
	PP	Casting	0.3	42
	PET	Casting	0.4	22
Nonwoven	Cellulose	Spunlace	2.0	26
	PET	Spunbond	1.4	20

**Table 2:** Description of specimens.

Specimen Code	Polymer	Substrate	Etching	Chemical Treatment
Cel	Cellulose	Flat film	None	None
Cel_-f_				C_4_F_8_ plasma, f
Cel_-Si_				DTMS deposition, Si
PP	PP			None
PET	PET			None
PET_-f_				C_4_F_8_ plasma, f
PET_-Si_				DTMS deposition, Si
Cel(SL)	Cellulose	Spunlace (SL) nonwoven		None
Cel(SL)_-f_				C_4_F_8_ plasma, f
Cel(SL)_-Si_				DTMS deposition, Si
PET(SB)	PET	Spunbond (SB) nonwoven		None
PET(SB)_-f_				C_4_F_8_ plasma, f
PET(SB)_-Si_				DTMS deposition, Si
PET(SB-etch)	PET		Alkali etching	None
PET(SB-etch)_-f_				C_4_F_8_ plasma, f
PET(SB-etch)_-Si_				DTMS deposition, Si

**Table 3:** Reference values of surface energy components of liquids [49].

Liquid	$\gamma_{L}$ (mN/m)	$\gamma_{L}^{d}$ (mN/m)	$\gamma_{L}^{p}$ (mN/m)	P_L_ (Polar Ratio)
Water (WA)	72.8	21.8	51.0	0.70
Formamide (FA)	58.4	31.4	27.0	0.46
Methylene iodide (MI)	50.8	50.4	0.40	0.008

Note: $\gamma_{L}$, overall surface energy of liquid; $\gamma_{L}^{d}$, dispersive component surface energy of liquid; $\gamma_{L}^{p}$, polar component surface energy of liquid; P_L_, ratio of the polar component to the overall surface energy of liquid.

**Table 4:** Contact angle of water and methylene iodide, and the estimated surface energy.

Specimen	Contact Angle (˚)		Surface Energy (mN/m)			Polar Ratio (P_S_)
	WA	MI	γ	γ^d^	γ^p^	
Cel	0	50	73.4	28.0	45.4	0.628
Cel_-f_	107	94	12.5	10.2	2.3	0.184
Cel_-Si_	101	60	28.6	28.2	0.42	0.015
PP	105	63	26.9	26.7	0.16	0.006
PET	96	61	28.5	27.2	1.34	0.047
PET_-f_	106	95	12.5	9.75	2.73	0.219
PET_-Si_	104	61	28.0	27.8	0.17	0.006

**Table 5:** XPS atomic concentration (%) of surfaces.

Specimen	C (%)	O (%)	F (%)	Si (%)
PP	97.8	2.2	-	-
Cel	77.1	22.9	-	-
Cel_-f_	52.0	30.9	17.2	
Cel_-Si_	51.2	27.3	-	21.6
PET	91.9	2.2	-	-
PET_-f_	49.2	7.3	43.5	-
PET_-Si_	67.8	23.3	-	8.8

**Table 6:** Contact and shedding angles of surfaces.

	CA (°)						ShA (°)	
Specimen	WA		FA		MI		WA	
Cel	0	(±0.0)	0	(±0.0)	50	(±0.8)	>50	(±0.0)
Cel_-f_	107	(±1.9)	105	(±1.6)	94	(±3.0)	>50	(±0.0)
Cel_-Si_	101	(±1.4)	84	(±2.8)	60	(±1.3)	>50	(±0.0)
PP	105	(±3.7)	88	(±2.3)	63	(±2.3)	>50	(±0.0)
PET	96	(±4.2)	82	(±1.9)	61	(±1.7)	>50	(±0.0)
PET_-f_	106	(±1.2)	102	(±2.6)	95	(±2.7)	>50	(±0.0)
PET_-Si_	104	(±2.3)	90	(±2.8)	61	(±1.9)	>50	(±0.0)
Cel(SL)	0	(±0.0)	0	(±0.0)	0	(±0.0)	>50	(±0.0)
Cel(SL)_-f_	166	(±2.3)	159	(±2.5)	153	(±3.4)	12.8	(±0.8)
Cel(SL)_-Si_	164	(±4.1)	152	(±2.3)	148	(±4.3)	8.8	(±0.8)
PET(SB)	126	(±2.8)	84	(±4.5)	0	(±0.0)	>50	(±0.0)
PET(SB)_-f_	147	(±2.9)	143	(±2.7)	140	(±3.7)	15.8	(±0.8)
PET(SB)_-Si_	145	(±3.8)	136	(±1.7)	127	(±2.5)	>50	(±0.0)
PET(SB-etch)	129	(±3.5)	0	(±0.0)	0	(±0.0)	>50	(±0.0)
PET(SB-etch)_-f_	156	(±2.9)	150	(±1.4)	150	(±2.4)	5.6	(±0.5)
PET(SB-etch)_-Si_	151	(±3.3)	145	(±3.2)	140	(±3.7)	7.0	(±0.6)

Note: shedding angle higher than 50° was not measured any further.
